# Atypical Chest Pain: A Diagnostic Challenge for Takotsubo Cardiomyopathy

**DOI:** 10.7759/cureus.105044

**Published:** 2026-03-11

**Authors:** Lisa Marie Babiak, Khachig K Ishkhan

**Affiliations:** 1 Clinical Sciences, Saint James School of Medicine, Chicago, USA; 2 Interventional Cardiology, Community First Medical Center, Chicago, USA

**Keywords:** broken heart syndrome, stress cardiomyopathy, stress-induced cardiomyopathy, takotsubo cardiomyopathy, takotsubo syndrome

## Abstract

Takotsubo cardiomyopathy (TTC) typically presents in post-menopausal females with chest pain, dyspnea, or palpitations and often mimics acute coronary syndrome. This case report describes a unique presentation of TTC in an elderly patient who exhibited gastrointestinal and musculoskeletal symptoms without cardiopulmonary complaints. This constellation of symptoms has not been previously described in the literature. The patient was ultimately diagnosed with TTC with evidence of ST-T wave abnormalities on ECG, elevated serum troponin levels, a reduced left ventricular ejection fraction, the absence of significant coronary artery disease on cardiac catheterization, and regional wall abnormalities on transthoracic ultrasound and left ventriculography. It contributes to our current understanding of the varied clinical presentation seen in TTC, which may help clinicians refine their differential diagnoses, provide earlier treatment, prevent misdiagnosis, and enhance patient care outcomes.

## Introduction

Takotsubo cardiomyopathy (TTC), commonly referred to as stress cardiomyopathy, involves transient left ventricular systolic dysfunction in the absence of obstructive coronary artery disease (CAD). First described in Japan in 1990, TTC is known to mimic acute coronary syndrome (ACS) and accounts for 1-2% of patients with elevated troponin levels [1-4]. It occurs most regularly in post-menopausal women and is frequently precipitated by emotional or physical stress [2,5-11]. Despite its reversible nature, TTC has been associated with potentially serious complications and mortality rates comparable to those seen in ST-segment elevation myocardial infarction (STEMI) [4,6,8,10]. As such, prompt diagnosis and treatment are of utmost importance.

The most widely accepted diagnostic criteria were proposed by the Mayo Clinic and comprise transient hypokinesis, akinesis, or dyskinesis of the left ventricle that extends beyond the territory supplied by a single coronary artery and occurs in the presence of emotional or physical stress, absence of significant CAD, ST-elevation and/or T-wave inversion on ECG or elevated cardiac troponin serum levels, and the absence of myocarditis or pheochromocytoma [3,12,13]. Patients classically present with symptoms suggestive of ACS, including chest pain, dyspnea, syncope, and palpitations [3,6,8,9].

There are currently few published case reports of patients with atypical or non-cardiac presentations. Recent studies have detailed patients with unilateral shoulder pain, exertional retrosternal chest pain, altered mental status, vomiting and diarrhea, facial droop, slurred speech, seizures, dyspnea with wheezing and sputum production, fatigue, and extreme thirst [14-22]. These cases underscore the diagnostic challenges posed by TTC when hallmark symptoms are absent. Despite these additions to our overall understanding of the varied presentations of TTC, those dominated by gastrointestinal and musculoskeletal pain are exceedingly rare. A comprehensive literature search in UpToDate, PubMed, and Google Scholar did not identify any prior cases with a similar case presentation.

## Case presentation

A 72-year-old Hispanic female with a past medical history of hypertension, hyperlipidemia, gastritis, hiatal hernia, and GERD presented to the emergency department with decreased appetite, generalized abdominal pain, and intermittent atraumatic lower back pain of one week's duration. During this period, she experienced approximately 10 episodes of nausea, non-bilious vomiting, and non-bloody diarrhea. She denied any fever, chest pain, shortness of breath, light-headedness, or dizziness. Her daughter reported mild alterations in the patient’s mental status but was unable to identify an inciting event. The patient affirmed adherence to her usual medications: metoprolol tartrate 25 mg, simvastatin 40 mg, and pantoprazole 40 mg daily.

**Figure 1:**
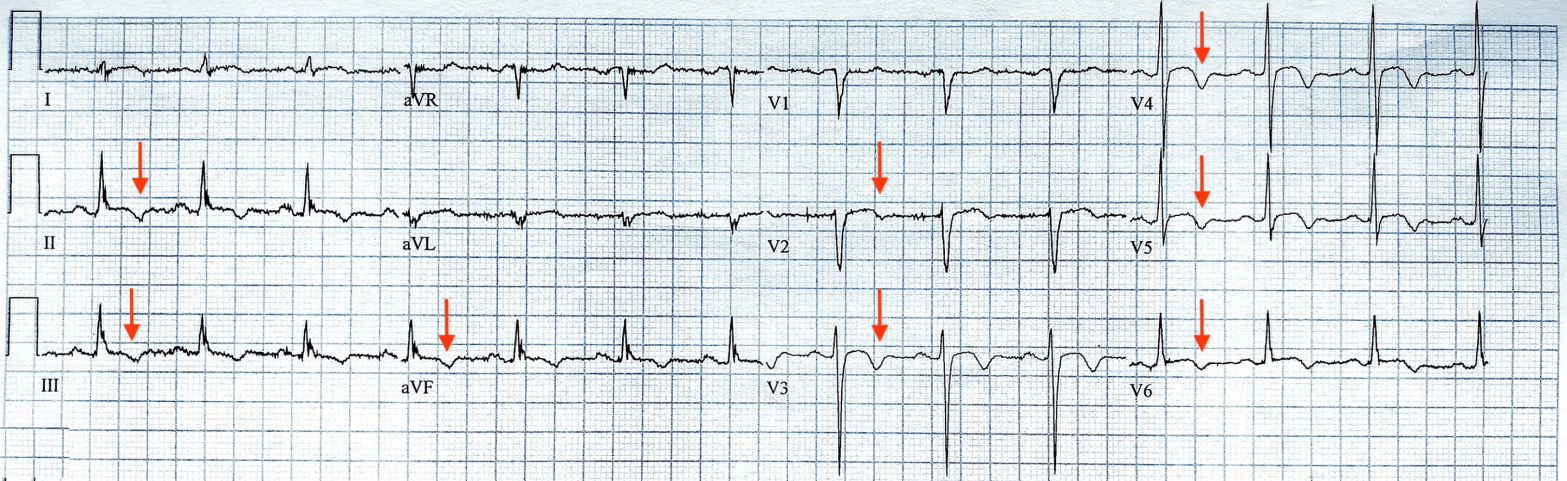
Electrocardiogram on arrival with diffuse ST-T abnormalities and T-wave inversions in leads II, III, aVF, and V2-V6 (red arrows).

On arrival, her vital signs were stable with a temperature of 36.9°C, blood pressure of 131/82, heart rate of 97/min, and oxygen saturation of 95% on room air. Gastroenterology services were consulted, and an abdominal CT and ultrasound were unremarkable. Initial cardiac testing revealed cardiomegaly on CXR and an elevated troponin I level of 373 ng/L (reference range = 0-10 ng/L). An ECG demonstrated diffuse ST-T wave abnormalities with T-wave inversion (Figure 1), representing a significant change from previous tracings and concern for non-ST elevation myocardial infarction (NSTEMI).

**Figure 2:**
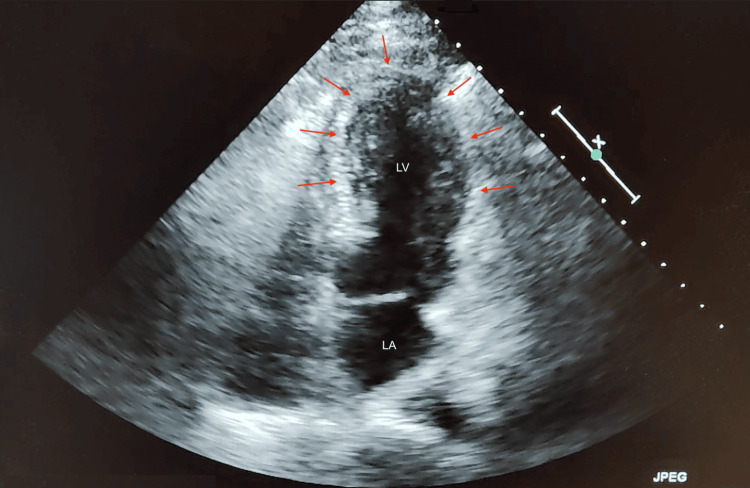
Echocardiogram without contrast medium in apical two-chamber view. Left ventricular (LV) systole showing hypokinesis of the mid and apical segments (red arrows).

**Figure 3:**
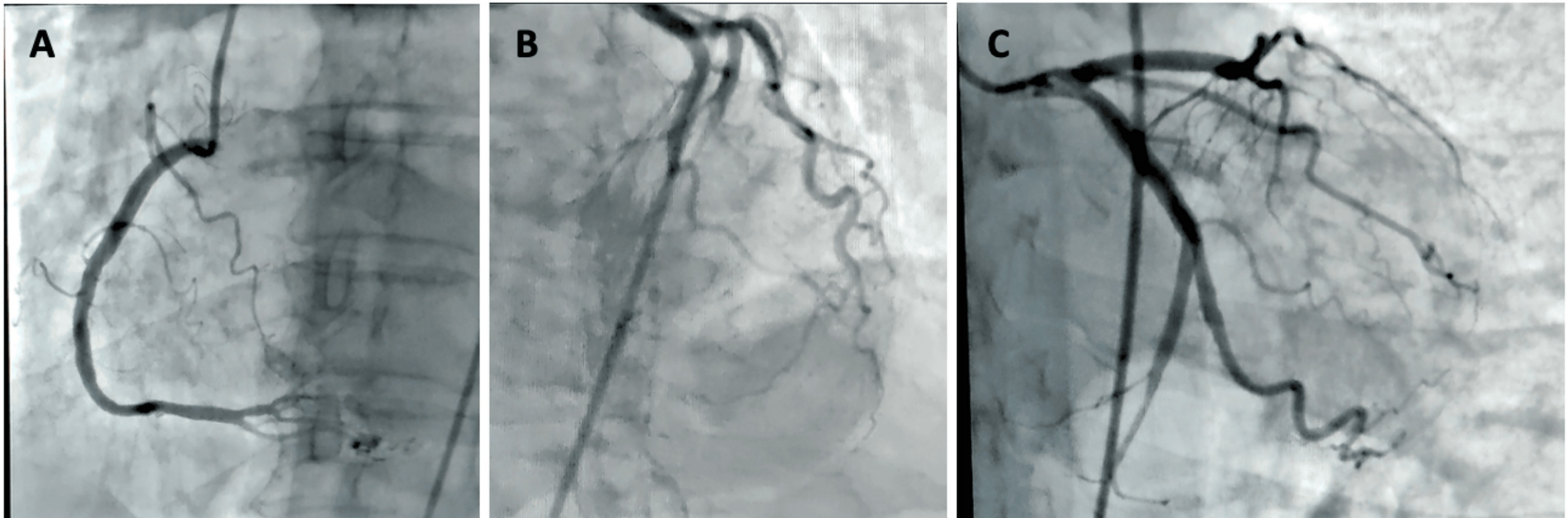
Coronary angiography revealed (A) right coronary artery without evidence of obstructive coronary artery disease, (B) left anterior descending artery without evidence of obstructive coronary artery disease, and (C) left circumflex and obtuse marginal branch without flow-limiting disease.

**Figure 4:**
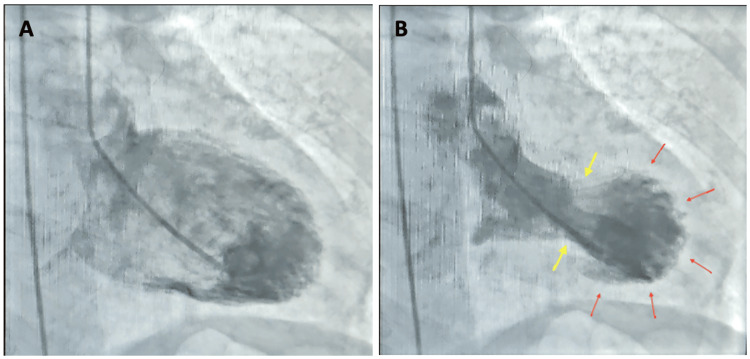
Left ventriculography. (A) End-diastolic image. (B) End-systolic image showing hyperkinesis of the left ventricular base (yellow arrows) and hypokinesis of the mid and apical segments (red arrows).

Cardiology services were consulted and recommended initiation of intravenous unfractionated heparin. A markedly prolonged partial thromboplastin time of 181 seconds was obtained, and heparin was held. Repeat troponin I measurement three hours later showed an upward trend to 497 ng/L, prompting scheduling of cardiac catheterization the following morning. Transthoracic echocardiogram (TTE) revealed a left ventricular ejection fraction (LVEF) of 24.53% and moderate anterior and septal wall hypokinesis of the mid and apical segments (Figure 2). Subsequent coronary angiography was performed with no evidence of significant coronary artery or flow-limiting disease (Figure 3). Left ventriculography revealed hypokinesis of the apical and mid segments (Figure 4) and confirmed the diagnosis of TTC.

Diagnosis was supported by the following: ECG findings showing diffuse ST-T wave abnormalities with T-wave inversion in leads II, III, aVF, and V2-V6, elevated cardiac enzymes (troponin I of 373 ng/L up trending to 497 ng/L), left heart catheterization with no evidence of significant CAD, and a LVEF of 24.53% with moderate anterior and septal wall hypokinesis of the mid and apical segments on TTE.

Further conversations with the patient and her daughter revealed the presence of pronounced emotional stress and anxiety due to the pending release and cohabitation of her incarcerated son.

Spironolactone and lisinopril were added to her treatment regimen. The patient remained hemodynamically stable throughout admission, and her hospital course was uncomplicated. She was transferred to the extended care unit on hospitalization day five and discharged after one week of hospitalization on aspirin 81 mg once daily, atorvastatin 40 mg once daily, lisinopril 10 mg once daily, metoprolol succinate 25 mg twice daily, and spironolactone 25 mg once daily.

## Discussion

This case features an atypical presentation of TTC in an elderly Hispanic female whose predominant symptoms of atraumatic lower back pain, decreased appetite, generalized abdominal pain, nausea, emesis, and diarrhea were initially suggestive of a gastrointestinal etiology. While TTC is classically associated with chest pain, dyspnea, syncope, and palpitations, the absence of cardiopulmonary symptoms underscores the clinical challenges in recognizing and diagnosing TTC.

The underlying mechanisms of TTC are not completely understood. Current theories implicate coronary vasospasm, microcirculatory dysfunction, and catecholamine-mediated myocardial stunning, which are believed to contribute to the transient regional systolic impairment and left ventricular ballooning that is pathognomonic of the syndrome [12,13]. Excess sympathetic stimulation during periods of emotional or physical stress is thought to result in neurohormonal activation and an associated surge in catecholamines and neuropeptide Y [12,13,23,24]. These mediators exert toxic effects on the myocardium, resulting in myocardial stunning [11-13,23,25]. Autonomic dysregulation may also influence gastrointestinal motility through effects on the brain-gut axis [25]. The catecholamine-mediated theory may contribute to the unique gastrointestinal symptoms observed in this case. This patient disclosed substantial emotional distress related to the pending release and return of her incarcerated son, which is consistent with the described associations between acute and chronic psychological triggers and the development of TTC [6,8,11].

This case highlights the importance of evidence on racial and socioeconomic disparities in the clinical presentation and outcome of patients diagnosed with TTC [8,22,26]. A study conducted by Ang et al. (2024) found that 35.9% of Hispanic patients diagnosed with TTC were also found to be living in the lowest median household income neighborhoods and experienced higher costs of hospitalization. Hispanic patients were associated with higher odds of developing dialysis-dependent acute kidney injury, as well as the highest inpatient mortality rates when compared to other racial groups [26]. These findings may reflect differences in the prevalence of comorbid conditions, including diabetes and obesity, among Hispanic populations and likely contribute to TTC’s varied clinical presentation.

This patient’s favorable hospital course is consistent with the current literature, noting an excellent prognosis of TTC with an in-hospital mortality rate of 4-8% and complete recovery in more than 90% of patients within four to eight weeks with supportive care [4,6-8,10,12,24]. The potential for serious complications, including arrhythmias, thromboembolism, and cardiogenic shock, requires clinicians to maintain diagnostic flexibility and vigilance in suspected ACS and TTC cases [6,7,10,12]. Increased awareness of the atypical presentations of TTC can reduce diagnostic delays and ultimately improve patient outcomes.

Cardiac magnetic resonance imaging (CMR) can play a valuable role in the diagnosis, management, and prognostication of TTC. It offers a comprehensive and non-invasive means of distinguishing TTC from acute myocardial infarction or myocarditis through the detection of myocardial edema and regional wall-motion abnormalities in the absence of irreversible myocardial injury [9,12,23,27,28]. A lack of late gadolinium enhancement on CMR has been associated with preserved myocardial viability and has been shown to reliably predict recovery of left ventricular function [23,27,28]. CMR was not performed in this case due to significant resource limitations at our safety-net hospital and the patient’s financial constraints. Diagnosis was therefore based on the available clinical and imaging data according to established diagnostic criteria proposed by the Mayo Clinic [3,12,13]. This case highlights how limited access to advanced cardiac imaging influences diagnostic evaluation in underserved populations.

Differentiating TTC from anterior STEMI continues to pose a clinical challenge due to their overlapping clinical presentations. The use of non-invasive tools, including ECG and speckle-tracking echocardiography (STE), has shown promising results in improving early diagnostic discrimination between these differential diagnoses. ECG provides one of the most accessible and timely tools in the evaluation of suspected ACS; however, proposed ECG patterns for distinguishing TTC from anterior STEMI lack sufficient sensitivity and specificity for standalone use [6,29]. Recent studies investigating the combined use of STE with ECG in the diagnosis and differentiation of TTC have found significantly lower LVEF and reduced global radial strain in patients with TTC compared to their STEMI counterparts [11,29].

## Conclusions

This case demonstrates the diagnostic challenges posed by TTC’s varied clinical presentations and the potential for delayed or missed diagnosis. In this patient, the predominance of gastrointestinal and musculoskeletal symptoms, in the absence of cardiopulmonary complaints, represents an undocumented presentation that may obscure early recognition of the condition. It further underscores the importance of considering TTC in atypical clinical scenarios. Continued research may reduce TTC-associated morbidity and mortality while also limiting the need for invasive procedures and unnecessary testing.

Despite diagnostic advances, the underlying pathophysiology of TTC remains unclear, and evidence-based treatment guidelines are still evolving. Prospective studies and controlled clinical trials are needed to clarify population-specific risk factors, establish standardized treatment protocols, and identify preventive strategies. Future research should focus on the potential role of strain analysis and ECG patterns in the diagnosis of TTC and its differentiation from anterior STEMI. Improved diagnostic accuracy and the development of standardized diagnostic scoring systems are essential for timely recognition, efficient management, and improved access to care.
